# Low disease activity for up to 3 years after adalimumab discontinuation in patients with early rheumatoid arthritis: 2-year results of the HOPEFUL-3 Study

**DOI:** 10.1186/s13075-017-1264-6

**Published:** 2017-03-14

**Authors:** Yoshiya Tanaka, Hisashi Yamanaka, Naoki Ishiguro, Nobuyuki Miyasaka, Katsuyoshi Kawana, Junko Kimura, Naoki Agata, Tsutomu Takeuchi

**Affiliations:** 1 The First Department of Internal Medicine, School of Medicine, University of Occupational and Environmental Health, Japan, 1-1 Iseigaoka, Yahatanishi-ku, Kitakyushu, Fukuoka 807-8555 Japan; 2 Institute of Rheumatology, Tokyo Women’s Medical University, 8-1, Kawada-cho, Shinjuku-ku, Tokyo, 162-8666 Japan; 3 Department of Orthopedic Surgery, Nagoya University Graduate School and School of Medicine, 65 Tsurumai-cho, Showa-ku, Nagoya, 466-8550 Japan; 4 Tokyo Medical and Dental University Graduate School, 1-5-45 Yushima, Bunkyo-ku, Tokyo, 113-8510 Japan; 5 AbbVie GK, 3-5-27, Mita, Minato-ku, Tokyo, 108-6302 Japan; 6 Division of Rheumatology, Department of Internal Medicine, School of Medicine, Keio University, 35 Shinanomachi, Shinjuku-ku, Tokyo, 160-0016 Japan

**Keywords:** Adalimumab, Biological agent, Observational study, Remission induction, Rheumatoid arthritis

## Abstract

**Background:**

This study was conducted to evaluate the feasibility of long-term adalimumab (ADA) discontinuation after achievement of low disease activity (LDA) in Japanese patients with early rheumatoid arthritis (RA) and to identify predictors of LDA maintenance.

**Methods:**

In the HOPEFUL-1 study, patients received initial therapy with either ADA plus methotrexate (MTX; intensive therapy) or MTX alone (standard therapy) for 26 weeks, followed by ADA + MTX for 26 weeks. In the HOPEFUL-2 study, patients received ADA + MTX (ADA continuation) or MTX alone (ADA discontinuation) for 52 weeks. HOPEFUL-3 was an observational study that enrolled patients who had completed HOPEFUL-2; these patients were followed for an additional 104 weeks.

**Results:**

Of the 172 patients enrolled in the HOPEFUL-3 study, 135 (ADA continuation, *n* = 61; ADA discontinuation, *n* = 74) with 28-joint Disease Activity Score using C-reactive protein (DAS28-CRP) values at both week 52 (start of HOPEFUL-2) and week 208 (end of HOPEFUL-3) were included in the effectiveness analysis. At week 208, 58 (95.1%) of 61 patients and 59 (79.7%) of 74 patients who continued or discontinued ADA, respectively, had LDA (DAS28-CRP <3.2). Initial intensive therapy was associated with a better outcome than standard therapy in terms of change in modified total Sharp score from week 0 to week 208, which was ≤0.5 (64% vs. 30%). The incidence of adverse events was significantly lower in the ADA discontinuation group than in the ADA continuation group (9.7% vs. 32.9%; *p* < 0.001).

**Conclusions:**

Approximately 80% of patients who discontinued ADA for 3 years after achieving LDA with ADA + MTX were still in LDA, with a lower incidence of adverse events than patients who continued ADA.

**Trial registration:**

ClinicalTrials.gov identifier: NCT01346501. Registered 29 April 2011.

## Background

Rheumatoid arthritis (RA) is a progressive inflammatory disease leading to joint destruction and functional disability [1, 2]. In the treatment of RA, the introduction of biological agents such as tumor necrosis factor (TNF) inhibitors has increased clinical remission rates and enabled long-term maintenance of structural and functional remission [3–5]. The results of several studies have shown that combination therapy with a TNF inhibitor and methotrexate (MTX) has more potent effects than MTX monotherapy [6–11]. However, the continuous use of biological agents is associated with an increased risk of serious infection [12] and may impose an economic burden on patients.

In recent studies, researchers have investigated the feasibility of discontinuing the use of biological agents in patients who have achieved low disease activity (LDA). In the multinational, large-scale, randomized OPTIMA trial, in patients with early RA (duration <1 year) who achieved LDA (28-joint Disease Activity Score based on C-reactive protein [DAS28-CRP] <3.2) by week 26 of treatment with adalimumab (ADA) in combination with MTX (ADA + MTX), the outcomes at week 78 in patients who discontinued ADA at week 26 were similar to those in patients who continued to receive ADA [11]. In Japan, the HONOR study was conducted to investigate the feasibility of ADA discontinuation for 1 year in patients receiving ADA + MTX for the treatment of established RA; 79% of patients with deep remission (defined as 28-joint Disease Activity Score based on erythrocyte sedimentation rate [DAS28-ESR] ≤1.98) were able to discontinue ADA without disease flare (defined as DAS28-ESR ≥3.2), which was similar to the proportion of patients without disease flare in the group that continued to receive ADA [13]. These results suggest that the achievement of disease control with initial intensive therapy may be necessary for the prevention of disease flare after discontinuation of ADA. This finding is useful in clinical practice; however, the effects of ADA discontinuation over a longer time period (i.e., >1 year) remain unknown.

Therefore, we conducted the HOPEFUL-3 study, a follow-up to the HOPEFUL-1 and HOPEFUL-2 studies [14, 15]. HOPEFUL-1 was designed to compare the effectiveness of ADA + MTX (ADA 40 mg every other week plus MTX 6–8 mg weekly) with MTX alone (6–8 mg weekly) for 26 weeks. Treatment with ADA + MTX was well-tolerated and effective in terms of improving clinical and radiographic responses in MTX-naive Japanese patients with early RA (duration <2 years), poor prognostic factors for RA, and high disease activity [14]. HOPEFUL-2 was the subsequent 52-week observational study that was carried out to evaluate the effects of ADA discontinuation on RA disease activity. Most patients who discontinued ADA were still in LDA for 1 year. Although the proportion of patients who were in LDA at 1 year in the ADA discontinuation group was significantly lower than in the continuation group (80% vs. 97%; *p* = 0.001), most patients maintained LDA in both groups. It was suggested that DAS28-CRP remission at the beginning of HOPEFUL-2 might be a predictor for LDA in patients discontinuing ADA [15]. In the present HOPEFUL-3 study, we observed patients with RA who had completed HOPEFUL-2 to assess the long-term effects of ADA discontinuation in terms of the proportion of patients without disease flare (defined as DAS28-CRP ≥3.2), as well as other measures of RA disease activity and safety.

## Methods

### Study design

**Fig. 1:**
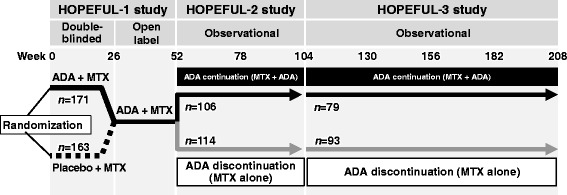
Study design for the HOPEFUL-1, HOPEFUL-2, and HOPEFUL-3 studies. *ADA* Adalimumab, *MTX* Methotrexate

HOPEFUL-3 was a 104-week observational study conducted between February 2011 and June 2014 in which patients enrolled in the HOPEFUL-1 and HOPEFUL-2 studies were followed (Fig. 1) [14, 15]. In brief, HOPEFUL-1 was a randomized, double-blind, placebo-controlled study comparing ADA + MTX with placebo + MTX for 26 weeks followed by an open-label ADA + MTX phase for all patients for 26 weeks. Eligible criteria were early RA (duration <2 years), high disease activity, poor prognostic factors, and no history of treatment with MTX. HOPEFUL-2 was an observational study of patients who agreed to an additional 1 year of data collection at the completion of HOPEFUL-1 (at week 52). The decision to discontinue ADA was made on the basis of patient preference after discussion with the patients’ doctors at the time of provision of their informed consent. In the ADA continuation group, patients were treated with ADA 40 mg and MTX, whereas patients in the ADA discontinuation group were treated with MTX alone. The analysis set consisted of patients who achieved DAS28-CRP <3.2 at weeks 46 and 52 in order to compare the outcomes between both groups. In HOPEFUL-3, patients with early RA who had completed the 1-year observational period of HOPEFUL-2 without receiving a biological agent other than ADA and who had provided informed consent to participate in this study were enrolled. During the 104-week HOPEFUL-3 study period, patients received either ADA + MTX (ADA continuation group) or MTX alone (ADA discontinuation group). This study was conducted in compliance with the Declaration of Helsinki and good postmarketing study practice in Japan, and it is registered with ClinicalTrials.gov (NCT01346501).

### Assessment of effectiveness

The primary endpoints were changes in DAS28-CRP and the proportion of patients who achieved LDA (DAS28-CRP < 3.2) at week 208. The secondary endpoints were the proportion of patients who achieved clinical remission (DAS28-CRP <2.6), and changes in Health Assessment Questionnaire Disability Index (HAQ-DI) [16] and modified total Sharp score (mTSS) [17] at week 208. Functional remission was defined as HAQ-DI ≤0.5, radiographic nonprogression was defined as a change in mTSS ≤0.5, and structural remission was defined as a change in mTSS (ΔmTSS) ≤0.5 per year. DAS28-CRP and HAQ-DI were also evaluated at weeks 104, 130, and 156, and radiographic findings of the hands and feet were also evaluated at weeks 104 and 156. To identify factors affecting effectiveness, we compared the results between patients who had received initial ADA + MTX (intensive therapy) and those who had received MTX alone (standard therapy) in HOPEFUL-1, and between patients who had continued to receive ADA and those who had discontinued ADA in HOPEFUL-2.

### Assessment of safety

All adverse events during HOPEFUL-3 were recorded and coded according to the Medical Dictionary for Regulatory Activities, version 18.1. An adverse event was considered a serious adverse event if it met any of the following criteria: death of the patient, life-threatening adverse event, hospitalization, prolongation of hospitalization, congenital anomaly, persistent or significant disability or incapability, significant medical event requiring medical or surgical intervention to prevent a serious outcome, spontaneous abortion, and elective abortion.

### Statistical analysis

The effectiveness analysis set included data from patients who were included in the analysis set of HOPEFUL-2 (DAS28-CRP <3.2 at weeks 46 and 52). The safety analysis set included data from all patients. Patient characteristics at 52 weeks were analyzed using Fisher’s exact test or the chi-square test for categorical variables and the Wilcoxon rank-sum test for continuous variables. Univariate logistic regression was used to identify predictors of LDA maintenance in the ADA discontinuation group. Multivariate regression was used to analyze factors with *p* < 0.1 in the univariate analyses. ROC curve analysis was used to explore cutoff values of the predictors.

Radiographic progression and functional outcomes over time were compared using the Wilcoxon signed-rank test. To handle missing values for the effectiveness analysis, last observation carried forward (LOCF) imputed values were used for clinical or functional outcomes, and linearly imputed values were used for mTSS.

Fisher’s exact test was used to compare the total number of adverse events, serious adverse events, and infections between the ADA continuation and discontinuation groups. *p* < 0.05 was considered significant. All analyses were carried out using StatView for Windows version 5.0 (Microsoft, Redmond, WA, USA) and SAS 9.3 (SAS Institute Inc., Cary, NC, USA) software.

## Results

### Patients

A total of 172 patients were enrolled in the HOPEFUL-3 study: 79 patients in the ADA continuation group and 93 patients in the ADA discontinuation group. Data from 135 patients (61 and 74 patients, respectively) were included in the effectiveness analysis set. Because the LOCF method was used, data from patients with missing DAS28-CRP values at weeks 130, 156, 182, or 208 were included if data after ADA discontinuation were available.

The safety analysis set included data from all 172 patients enrolled. Table 1 summarizes the baseline characteristics of patients whose data was used in the effectiveness analysis. The swollen 28-joint count was higher in the ADA continuation group at week 52 (*p* < 0.01). In both the ADA continuation group and the ADA discontinuation group, the joint space narrowing score and mTSS at week 52 were higher in patients who had received initial MTX monotherapy than in those who had received initial ADA + MTX (*p* = 0.04 and *p* = 0.01, and *p* = 0.04 and *p* = 0.02, respectively).

**Table 1:** Patient characteristics at baseline (week 52, the start of HOPEFUL-2) in the effectiveness analysis set (*n* = 135)^a^

	ADA continuation group (*n* = 61)	ADA discontinuation group (*n* = 74)	*p* Value^b^	ADA continuation group		*p* Value^b^	ADA discontinuation group		*p* Value^b^
				Intensive therapy (ADA + MTX) (*n* = 28)	Standard therapy (MTX alone) (*n* = 33)		Intensive therapy (ADA + MTX) (*n* = 37)	Standard therapy (MTX alone) (*n* = 37)	
Female sex, *n* (%)	52 (85.2)	61 (82.4)	0.82	25 (89.3)	27 (81.8)	0.49	33 (89.2)	28 (75.7)	0.22
Age, years	58.3 ± 13.6	54.5 ± 12.4	0.06	59.5 ± 12.5	57.3 ± 14.5	0.58	53.4 ± 11.2	55.5 ± 13.6	0.41
Disease duration, years	1.3 ± 0.4	1.2 ± 0.3	0.35	1.3 ± 0.3	1.3 ± 0.5	0.46	1.2 ± 0.3	1.3 ± 0.3	0.20
MTX dose, mg/week	6.9 ± 1.8	7.1 ± 1.9	0.51	6.5 ± 1.4	7.3 ± 2.0	0.03^c^	7.0 ± 2.0	7.3 ± 1.8	0.19
Steroid use, *n* (%)	17 (27.9)	17 (23.0)	0.55	7 (25.0)	10 (30.3)	0.78	5 (13.5)	12 (32.4)	0.10
TJC28 score	0.7 ± 1.1	1.0 ± 2.0	0.93	0.4 ± 0.7	0.9 ± 1.3	0.08	1.3 ± 2.4	0.8 ± 1.4	0.39
SJC28 score	1.0 ± 1.7	0.4 ± 0.9	0.006^d^	1.1 ± 1.6	1.0 ± 1.9	0.24	0.5 ± 1.0	0.3 ± 0.8	0.57
CRP, mg/dl	0.2 ± 0.3	0.1 ± 0.2	0.93	0.2 ± 0.2	0.2 ± 0.3	0.10	0.1 ± 0.1	0.1 ± 0.2	0.33
ESR, mm/h	18.8 ± 14.2	18.3 ± 13.0	0.88	18.0 ± 13.1	19.5 ± 15.3	0.90	19.4 ± 12.5	17.2 ± 13.5	0.23
EGA score (VAS, mm)	8.2 ± 8.8	7.6 ± 7.1	0.81	8.8 ± 9.5	7.7 ± 8.3	0.73	8.1 ± 7.5	7.1 ± 6.7	0.73
PGA score (VAS, mm)	9.0 ± 9.0	8.5 ± 11.0	0.27	6.8 ± 7.1	10.8 ± 10.1	0.08	7.7 ± 8.9	9.2 ± 12.9	0.46
Pain score (VAS, mm)	8.2 ± 7.5	8.5 ± 10.5	0.56	6.8 ± 7.3	9.4 ± 7.6	0.13	7.6 ± 7.6	9.4 ± 12.7	0.51
DAS28-ESR	2.5 ± 0.8	2.4 ± 0.8	0.42	2.4 ± 0.6	2.6 ± 0.8	0.28	2.5 ± 0.8	2.2 ± 0.8	0.08
DAS28-CRP	1.8 ± 0.5	1.7 ± 0.6	0.23	1.8 ± 0.5	1.9 ± 0.5	0.35	1.8 ± 0.6	1.7 ± 0.6	0.52
HAQ-DI score	0.193 ± 0.273	0.199 ± 0.303	0.68	0.125 ± 0.167	0.250 ± 0.329	0.20	0.213 ± 0.296	0.186 ± 0.313	0.57
Erosion score	6.0 ± 7.7	7.1 ± 9.7	0.08	4.3 ± 4.8	7.3 ± 9.3	0.12	6.7 ± 12.3	7.5 ± 6.1	0.09
JSN score	3.4 ± 3.6	6.7 ± 13.1	0.31	2.2 ± 1.8	4.5 ± 4.4	0.04^c^	5.4 ± 15.1	8.1 ± 10.8	0.01^c^
mTSS	9.4 ± 10.4	13.9 ± 21.9	0.09	6.5 ± 5.9	11.8 ± 12.6	0.04^c^	12.1 ± 27.2	15.6 ± 15.0	0.02^c^
MMP-3 (ng/ml)	55.2 ± 44.1	60.7 ± 48.1	0.63	52.7 ± 31.9	57.4 ± 52.6	0.80	65.5 ± 49.9	56.0 ± 46.5	0.21
RF (U/ml)	62.8 ± 90.8	42.6 ± 51.8	0.85	50.6 ± 94.1	73.1 ± 88.1	0.11	41.8 ± 35.3	43.4 ± 64.8	0.26
ACPA (U/ml)	169.3 ± 257.8	216.2 ± 654.8	0.41	116.0 ± 255.0	214.6 ± 255.1	0.02^c^	167.6 ± 285.4	264.8 ± 885.0	0.10

*Abbreviations: ACPA* Anti-cyclic citrullinated peptide antibody, *ADA* adalimumab, *CRP* C-reactive protein, *DAS28-CRP* 28-joint Disease Activity Score based on C-reactive protein, *DAS28-ESR* 28-joint Disease Activity Score based on erythrocyte sedimentation rate, *EGA* Evaluator global assessment, *ESR* Erythrocyte sedimentation rate, *HAQ-DI* Health Assessment Questionnaire Disability Index, *JSN* Joint space narrowing, *MMP-3* Matrix metalloproteinase 3, *mTSS* Modified total Sharp score, *MTX* Methotrexate, *PGA* Patient global assessment, *RF* Rheumatoid factor

^a^ Values are expressed as mean ± SD, unless otherwise indicated

^b^ Statistical significance assessed by the Wilcoxon rank sum test

^c^ *p* < 0.05 ADA continuation group versus ADA discontinuation group, or initial intensive therapy (ADA + MTX) group versus standard therapy (MTX alone) group

^d^ *p* < 0.01 ADA continuation group versus ADA discontinuation group, or initial intensive therapy (ADA + MTX) group versus standard therapy (MTX alone) group

### Changes in DAS28-CRP and sustainability of LDA

**Fig. 2:**
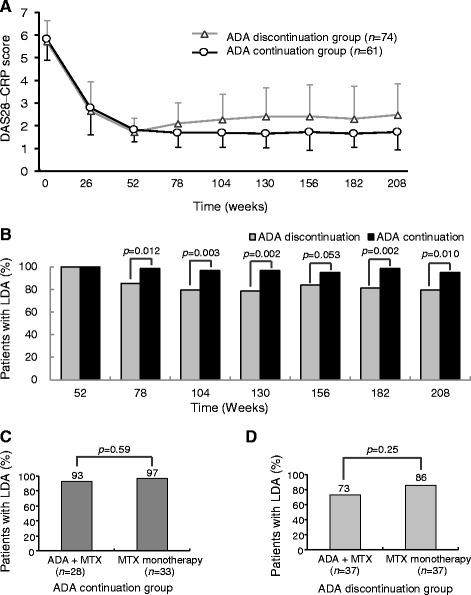
Changes in DAS28-CRP (mean ± SD) over time in patients whose data were used in the analysis of effectiveness (*n* = 135) (**a**). Proportion of patients with LDA during the study period (**b**). Comparison of proportion of patients with LDA at the end of the HOPEFUL-3 study period (week 208) according to the initial therapy in the ADA continuation group (**c**) and in the ADA discontinuation group (**d**). *ADA* Adalimumab, *DAS28-CRP* 28-joint Disease Activity Score based on C-reactive protein, *LDA* Low disease activity, *MTX* Methotrexate

Changes in mean DAS28-CRP values in the ADA continuation group and ADA discontinuation group are shown in Fig. 2a. At week 52, the start of the HOPEFUL-2 study, scores were similar in both groups. After week 52, DAS28-CRP values in patients who had discontinued ADA increased slightly until week 130 and remained stable thereafter; in patients who had continued to receive ADA, DAS28-CRP values remained stable until week 208.

In the ADA discontinuation group, the proportion of patients with LDA decreased to about 80% by week 104 (the start of the HOPEFUL-3 study) and remained unchanged thereafter; in contrast, only a slight decrease was observed in the ADA continuation group. At week 208 (the end of the HOPEFUL-3 study), 59 (79.7%) of the 74 patients in the ADA discontinuation group were in LDA, although this was significantly lower than the corresponding value in the ADA continuation group (i.e., 58 [95.1%] of 61 patients; *p* = 0.010) (Fig. 2b). The persistence of LDA did not differ according to the initial therapy (ADA + MTX vs. MTX monotherapy) in either the ADA continuation group (Fig. 2c) or the ADA discontinuation group (Fig. 2d).

### Factors affecting sustainability of LDA

Among patient characteristics at week 52 in the ADA discontinuation group, CRP, ESR, DAS28-ESR, rheumatoid factor (RF), and anti-cyclic citrullinated peptide antibody values were significantly different between patients who were in LDA and those who failed to be in LDA at week 208 (Table 2). Multivariate regression analysis showed that DAS28-CRP and RF at week 52 significantly affected the sustainability of LDA from week 52 to week 208. For RF, subsequent ROC curve analysis identified a cutoff value of 87.0 U/ml (sensitivity, 94.9%; 1-specificity, 60.0%). LDA at week 208 was reported in 86.2% (56/65) and 33.3% (3/9) of patients with RF values ≤87 and > 87, respectively, with a significant difference between the two groups (*p* = 0.0016). For DAS28-CRP, 1.40 was identified as a cutoff value (sensitivity, 47.5%; 1-specificity, 20.0%); no significant difference was observed between the two groups (*p* = 0.0787).

**Table 2:** Patient characteristics at week 52 by low disease activity at week 208 in the adalimumab discontinuation group^a^

	LDA at week 208 (*n* = 59)		Failed LDA at week 208 (*n* = 15)		*p* Value ^b^
	Mean	SD	Mean	SD	
Female sex, *n* (%)	49	83.1	12	80.0	1.0000
Age, years	54.1	11.5	55.8	15.9	0.4675
Disease duration, years	1.3	0.3	1.2	0.3	0.4079
MTX dose, mg/week	7.3	2.0	6.5	1.6	0.2432
Steroid use, *n* (%)	14	23.7	3	20.0	1.0000
TJC28	0.9	2.0	1.3	1.9	0.2210
SJC28	0.4	0.9	0.4	0.9	0.9329
CRP, mg/dl	0.1	0.2	0.2	0.1	0.0026^c^
ESR, mm/h	16.4	11.2	25.9	16.7	0.0154^d^
EGA	7.2	6.9	9.1	8.0	0.4145
PGA	7.2	8.4	13.5	17.5	0.1600
Pain VAS	7.5	7.8	12.6	17.2	0.3242
DAS28-ESR	2.3	0.7	2.8	0.9	0.0113^d^
DAS28-CRP	1.7	0.6	2.0	0.7	0.0715
HAQ	0.197	0.298	0.208	0.330	0.9240
Erosion	6.5	5.4	9.7	18.9	0.7467
JSN	6.3	9.1	8.3	23.5	0.1199
mTSS	12.8	12.8	18.0	42.2	0.2390
MMP-3, ng/ml	59.0	47.4	67.8	51.9	0.2475
RF	32.3	33.0	83.1	85.3	0.0077^c^
ACPA, U/ml	113.4	237.9	620.5	1334.9	0.0113^d^

*Abbreviations: ADA* Adalimumab, *LDA* Low disease activity, *MTX* Methotrexate, *TJC* Tender joint count, *SJC* Swollen joint count, *CRP* C-reactive protein, *ESR* Erythrocyte sedimentation rate, *EGA* Evaluator global assessment, *PGA* Patient global assessment, *VAS* Visual analogue scale, *DAS28* 28-joint Disease Activity Score, *HAQ* Health Assessment Questionnaire, *JSN* Joint space narrowing, *mTSS* Modified total Sharp score, *MMP-3* Matrix metalloproteinase 3, *RF* Rheumatoid factor, *ACPA* Anti-cyclic citrullinated peptide antibody

^a^ Values are expressed as mean ± SD, unless otherwise indicated

^b^ Statistical significance assessed by Fisher’s exact test for categorical data and the Wilcoxon rank sum test for continuous data

^c^ *p* < 0.01 LDA vs. failed LDA at week 208

^d^ *p* < 0.05 LDA vs. failed LDA at week 208

### Clinical and functional remission

**Fig. 3:**
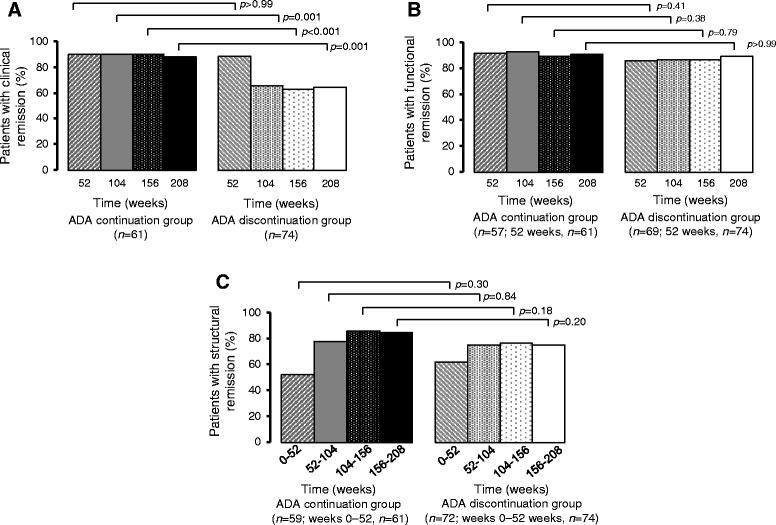
Proportion of patients who achieved **a** clinical remission, defined as DAS28-CRP <2.6, **b** functional remission, defined as HAQ-DI ≤0.5, at weeks 52, 104, 156, and 208, and **c** structural remission, defined as ΔmTSS ≤0.5 per year, within specified 52-week periods. *ADA* Adalimumab, *DAS28-CRP* 28-joint Disease Activity Score based on C-reactive protein, *HAQ-DI* Health Assessment Questionnaire Disability Index, *mTSS* Modified total Sharp score, *ΔmTSS* Change in modified total Sharp score

The proportions of patients who achieved clinical remission were 66.0%, 64.0%, and 65% at weeks 104, 156, and 208, respectively, in the ADA discontinuation group; these values were significantly lower than the corresponding percentages in the ADA continuation group of 90%, 90%, and 89% at weeks 104, 156, and 208, respectively (*p* = 0.001, *p* < 0.001, and *p* = 0.001, respectively) (Fig. 3a). In contrast, the proportion of patients who achieved functional remission at weeks 104, 156, and 208 was similar between the two groups at about 90% (Fig. 3b).

### Structural remission

**Fig. 4:**
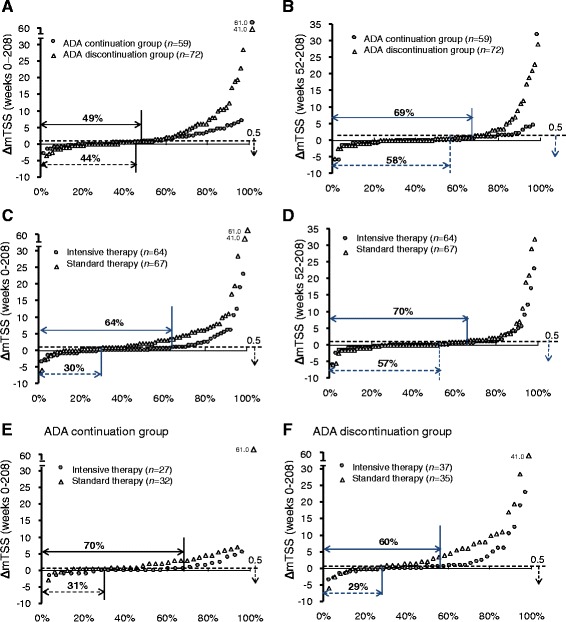
Cumulative probability plots showing ΔmTSS. Comparison of the ADA continuation group and the ADA discontinuation group over 4 years (weeks 0–208) (**a**) and over 3 years (weeks 52–208) (**b**); comparison of the initial intensive therapy group and the standard therapy group over 4 years (weeks 0–208) (**c**) and over 3 years (weeks 52–208) (**d**); and comparison of the initial intensive therapy group and the standard therapy group over 4 years (weeks 0–208) in the ADA continuation group (**e**) and in the ADA discontinuation group (**f**). *ADA* Adalimumab, *ΔmTSS* Change in modified total Sharp score

Radiographic progression was assessed on the basis of change in mTSS. There were no significant differences between the ADA discontinuation and continuation groups in the proportion of patients who achieved structural remission in respective 52-week periods (Fig. 3c). Radiographic nonprogression from week 0 to week 208 was achieved by 29 (49%) of 59 patients in the ADA continuation group and 32 (44%) of 72 patients in the ADA discontinuation group. Patients with radiographic progression (ΔmTSS >0.5) in the ADA discontinuation group had greater ΔmTSS compared with those in the ADA continuation group (Fig. 4a). Between weeks 52 and 208, 69% of patients in the ADA continuation group and 58% in the discontinuation group achieved ΔmTSS ≤0.5. Only one patient in the ADA continuation group experienced a change >15 in mTSS from week 52 to week 208, as compared with five patients in the ADA discontinuation group (Fig. 4b).

The proportion of radiographic nonprogression between weeks 0 and 208 was greater in patients who received initial intensive therapy with ADA + MTX than in patients who received initial standard therapy with MTX alone (64% of patients [41 of 64] vs. 30% of patients [20 of 67]) (Fig. 4c). The difference between these two groups was moderate between weeks 52 and 208, with 70% of patients in the initial intensive group and 57% in the discontinuation group achieving radiographic nonprogression (Fig. 4d).

The greater radiographic nonprogression rate in patients receiving initial intensive therapy than in those receiving initial standard therapy was continuously observed during the long-term follow-up period, and this rate was maintained even after week 52, regardless of subsequent continuation or discontinuation of ADA (70% vs. 31% and 60% vs. 29%, respectively) (Fig. 4e and f).

### Safety

Table 3 summarizes the adverse events reported in all patients enrolled in the HOPEFUL-3 study. During the study period (weeks 104–208), there were no cases of tuberculosis and no deaths. The adverse events that had been reported in more than two patients in the ADA continuation group were infections and infestations (8.9% [7 of 79 patients] and 2.2% [2 of 93 patients] in the ADA continuation and discontinuation groups, respectively), investigations (5.1% [4 of 79 patients] and 2.2% [2 of 93 patients], respectively), gastrointestinal disorders (3.8% [3 of 79 patients] and 3.2% [3 of 93 patients], respectively), and musculoskeletal and connective tissue disorders (3.8% [3 of 79 patients] and 1.1% [1 of 93 patients], respectively). The proportion of patients who experienced adverse events was significantly lower in the ADA discontinuation group than in the ADA continuation group (9.7% [9 of 93 patients] vs. 32.9% [26 of 79 patients]; *p* < 0.001). In the ADA continuation group, four serious adverse events occurred in three patients (3.8%): bronchiolitis, urinary tract stone, ascites fluid, and hepatic cirrhosis. One serious infection, bronchiolitis, was reported in one patient (1.3%). In the ADA discontinuation group, one patient had serious interstitial lung disease (1.1%).

**Table 3:** Summary of adverse events experienced by patients in the HOPEFUL-3 study (*n* = 172)^a^

	ADA continuation group (*n* = 79)	ADA discontinuation group (*n* = 93)	*p* Value^b^
Any adverse event	26 (32.9)	9 (9.7)	<0.001
Serious adverse event	3 (3.8)	1 (1.1)	0.33
Infectious adverse event	9 (11.4)	4 (4.3)	0.09
Serious infection	1 (1.3)	0 (0.0)	NA
Bronchiolitis	1 (1.3)	0 (0.0)	NA

*ADA* Adalimumab, *NA* Not applicable

^a^Values are expressed as *n* (%)

^b^Statistical significance as assessed by Fisher’s exact test

## Discussion

In the HOPEFUL-3 study, we investigated whether patients who achieved LDA would be in LDA at 3 years after discontinuation of ADA. The proportion of patients who achieved LDA at week 208 was significantly lower in the ADA discontinuation group than in the ADA continuation group. However, in each group, the majority of patients were in LDA at the end of the study period: 95.1% in the ADA continuation group and 79.7% in the ADA discontinuation group. These results are similar to the results of the HOPEFUL-2 study, in which LDA was achieved by 97.0% and 80.0% of patients in the ADA continuation group and the ADA discontinuation group, respectively [15]. The results of the HOPEFUL-3 study therefore showed that patients were in LDA after 2 years.

The early prediction of long-term clinical outcomes during a treatment course is of great value for physicians. As previously reported in the HOPEFUL-2 study, DAS28-CRP was identified as a factor associated with persistence of LDA [15]. The estimated cutoff value of DAS28-CRP using ROC analysis (1.40) identified in the HOPEFUL-3 study was lower than the value identified in the HOPEFUL-2 study (2.0). It is not surprising that deeper regression might be needed to maintain longer LDA. In the present study, RF was suggested as an additional possible predictive factor, but the result should be interpreted with caution because the patients were not randomized.

At the start of the HOPEFUL-2 study, there were no significant differences in clinical indexes between the two groups in the proportion of patients who achieved LDA, in either the ADA continuation group or the ADA discontinuation group. However, structural indexes (i.e., erosion score, joint space narrowing score, and mTSS) were significantly lower in the intensive therapy group than in the initial standard therapy group. The prevention or slowing of radiographic progression with initial intensive therapy with ADA + MTX may persist even after long-term ADA discontinuation. In order to achieve long-term suppression of joint destruction in patients with early RA, initial intensive therapy with ADA plus MTX might be considered as an effective therapeutic approach.

Although the use of biological agents leads to clinical, functional, and structural remission, continuous use of these drugs may lead to adverse events, particularly serious infection. In the HOPEFUL-3 study, there were no cases of tuberculosis and no deaths. Patients who continued to receive ADA were more likely to experience an adverse event than those who discontinued ADA. Less than 5% of patients who discontinued ADA had an infection, and none of the patients had a serious infection. In the HOPEFUL-2 study period (weeks 52–104), the proportions of patients who experienced adverse events in the ADA continuation group and the ADA discontinuation group were higher than in the same groups in the HOPEFUL-3 study period (weeks 104–208), with a smaller difference between the two groups (48.1% vs. 32.9%, respectively, in the HOPEFUL-2 study) [15]. The lower incidence of adverse events in the HOPEFUL-3 study may be explained by the long-term discontinuation of ADA.

In addition to the safety benefits for patients with RA, discontinuation of biological agents after achievement of LDA or remission may also benefit the healthcare system by reducing the economic burden associated with the long-term use of biological agents. Further studies are necessary to clarify the appropriate long-term therapeutic approach for the use of biological agents from a cost-benefit perspective.

At the end of the HOPEFUL-1 trial, patients could decide whether to enter this observational study. The participants were not re-randomized and could decide to continue or discontinue ADA. Potential differences in disease and treatment perception of patients and their doctors cannot be excluded. Thus, the results of this study were not conclusive, but they suggested that long-term discontinuation of ADA treatment might be a feasible and beneficial therapeutic option for patients with early RA who achieved LDA.

### Limitations

This study has several limitations. The study was observational in nature. In addition, patients were not randomly assigned to the treatment groups. Patients from different economic backgrounds were included in this study, so physicians may have taken this into account when deciding whether to continue ADA, resulting in a potential selection bias. There may be additional steroid use, which could have influenced the outcomes at reported time points; however, we do not have sufficient information regarding the additional steroid use to conduct analysis. Finally, because we assessed whether patients were in LDA at the end of the study period, we are unable to infer whether patients were in sustained remission throughout the study period.

## Conclusions

The results of this 104-week follow-up study indicated that approximately 80% of the Japanese patients with early RA were in LDA after 3 years ADA discontinuation. However, it must be noted that there was a significant difference in the proportion of patients who achieved LDA among patients who discontinued ADA compared with patients who continued to receive ADA. The initial therapy had no effect on LDA sustainability in either the ADA continuation group or the ADA discontinuation group. However, initial intensive therapy with ADA + MTX was associated with a better outcome in terms of suppression of joint destruction compared with standard therapy. As ADA discontinuation is associated with a lower incidence of adverse events, physicians should weigh the risks and benefits of ADA discontinuation.

## Abbreviations

ACPA: Anti-cyclic citrullinated peptide antibody
ADA: Adalimumab
CRP: C-reactive protein
DAS28: 28-joint Disease Activity Score
EGA: Evaluator global assessment
ESR: Erythrocyte sedimentation rate
HAQ-DI: Health Assessment Questionnaire Disability Index
JSN: Joint space narrowing
LDA: Low disease activity
LOCF: Last observation carried forward
MMP-3: Matrix metalloproteinase 3
mTSS: Modified total Sharp score
MTX: Methotrexate
PGA: Patient global assessment
RA: Rheumatoid arthritis
RF: Rheumatoid factor
SJC: Swollen joint count
TNF: Tumor necrosis factor
VAS: Visual analogue scale
